# What is the clinical utility of acoustic and vibrational analyses in uncemented total hip arthroplasty?

**DOI:** 10.1186/s42836-024-00280-0

**Published:** 2024-12-03

**Authors:** Shlok Patel, Christian J. Hecht, Yasuhiro Homma, Atul F. Kamath

**Affiliations:** 1 Department of Orthopaedic Surgery, B.J. Medical College, Ahmedabad, Gujarat 380016 India; 2 Department of Orthopaedic Surgery, Cleveland Clinic Foundation, Cleveland, OH 44195 USA; 3 Department of Medicine for Orthopaedics and Motor Organs, Juntendo University Graduate School of Medicine, Bunkyo-Ku, Tokyo, 113-8421 Japan

**Keywords:** Acoustics, Acoustic analysis, Fracture, Implant stability, Total hip arthroplasty

## Abstract

**Background:**

Despite recent developments in THA, a more objective method is needed to assist orthopedic surgeons in identifying the insertion endpoint of the broaching procedure. Therefore, this systematic review evaluated the in-vivo efficacy of various acoustic and vibration analyses in detecting proper implant seating, identifying intraoperative complications, and quantifying the accuracy of predictive modeling using acoustics.

**Methods:**

Four electronic databases were searched on July 23rd, 2023, to retrieve articles evaluating the use of acoustic analysis during THA. The search identified 835 unique articles, which were subsequently screened by two independent reviewers as per our inclusion and exclusion criteria. In total, 12 studies evaluating 580 THAs were found to satisfy our criteria and were included in this review.

**Results:**

Methodologically, analyses have suggested stopping broaching when consecutive blows emit similar acoustic profiles (maximum peak frequency ± 0.5 kHz), which indicates proper implant seating in terms of stability and mitigates subsidence. Also, abrupt large deviations from the typical progression of acoustic signals while broaching are indicative of an intraoperative fracture. Since height, weight, femoral morphological parameters, and implant type have been shown to alter acoustic emissions while hammering, incorporating these factors into models to predict subsidence or intraoperative fracture yielded virtually 100% accuracy in identifying these adverse events.

**Conclusion:**

These findings support that acoustic analyses during THA show promise as an accurate, objective, and non-invasive method to predict and detect proper implant fixation as well as to identify intraoperative fractures.

**Trial registration:**

PROSPERO registration of the study protocol: CRD42023447889, 23 July 2023.

## Introduction

Although total hip arthroplasty (THA) often successfully provides long-term relief of pain and improves joint function, concerns remain about periprosthetic or prosthetic fracture, subsidence, stem size mismatch, and instability, all of which may compromise the longevity of the implant [1]. Currently, precise intraoperative implant fixation relies solely on the intuitive judgment of the surgeon. Surgeons need to apply adequate force to achieve firm fixation; however, excessive force can lead to femur fractures, whereas insufficient force results in postoperative subsidence [2, 3] The critical point where the fixation is sufficient and the fracture risk is minimal is the insertion end point [4]. To ensure reaching the end point of insertion, the surgeon depends on experience, audible changes in the sound produced by hammering, and the tactile feel of a well-fitted implant [3, 5] The iatrogenic femur fractures in THAs, which cause protracted operative time, a larger incision, increased blood loss, and delayed postoperative recovery, occurred reportedly in 1% to 28% of THAs [6]. Consequently, a more objective method is needed to measure precise implant fixation and mitigate intraoperative and postoperative complications associated with mal-placement and fractures [7, 8]

Acoustic emission technology and vibrational analyses have shown promise to help surgeons identify the insertion end point, thereby contributing to better implant stability. Various biomechanical models, *in-vitro* studies, orthopedic models, and cadaveric studies have identified vibration-based methods as a potential non-destructive method to assess the progression of hip fixation in real-time [9–12]. Additionally, objective and quantitative analysis of hammering sounds during implant insertion has been utilized as a tool for implant fixation and fracture prediction in animal models, human cadavers, *in-vitro* setups, and in-vivo setups with variable success [1, 5]. Although multiple studies have demonstrated that acoustic analysis could potentially help surgeons identify the end-point during THA, the considerable heterogeneity in study design and outcomes assessed renders it difficult to make comparisons across studies [1]. Therefore, a systematic review of the available literature was indispensable to better understand the potential benefits of acoustic and vibrational analyses in THA, discern trends among studies with similar designs, evaluate methodological quality, inform clinical practices, and guide future research.

Specifically, this review tried to answer the following questions (1) Do vibrational and acoustic analyses accurately detect proper implant seating and subsidence as well as (2) intraoperative fractures for THA? (3) Do patient characteristics or implant type impact acoustic analyses? (4) Are predictive models generated via acoustic analyses for detecting adverse intraoperative and postoperative events accurate?

## Methods

### Query strategy

A search of Google Scholar, EBSCOhost, MEDLINE, and PubMed was conducted on July 23, 2023, to retrieve articles evaluating the use of acoustic analysis during THA. The “AND” and “OR” Boolean operators were used in conjunction with Medical Subject Headings (Mesh) to build the search term: “Arthroplasty, Replacement, Hip”[Mesh] OR “Arthroplasty, Replacement”[Mesh] OR “Hip Prosthesis”[Mesh] OR total hip arthroplasty OR THA AND “Acoustics”[Mesh] OR “Sound”[Mesh] OR “Vibration”[Mesh] OR “Microscopy, Acoustic”[Mesh] OR acoustic emission OR vibro-acoustic.

### Eligibility criteria

Articles were included if they had an English full-text manuscript published, and the study assessed the utility of acoustic or vibrational emission analyses during total hip arthroplasty. Articles were excluded if they were duplicates, published before January 1, 2000, meta-analyses or reviews, editorials, or pre-prints.

### Study selection

**Fig. 1:**
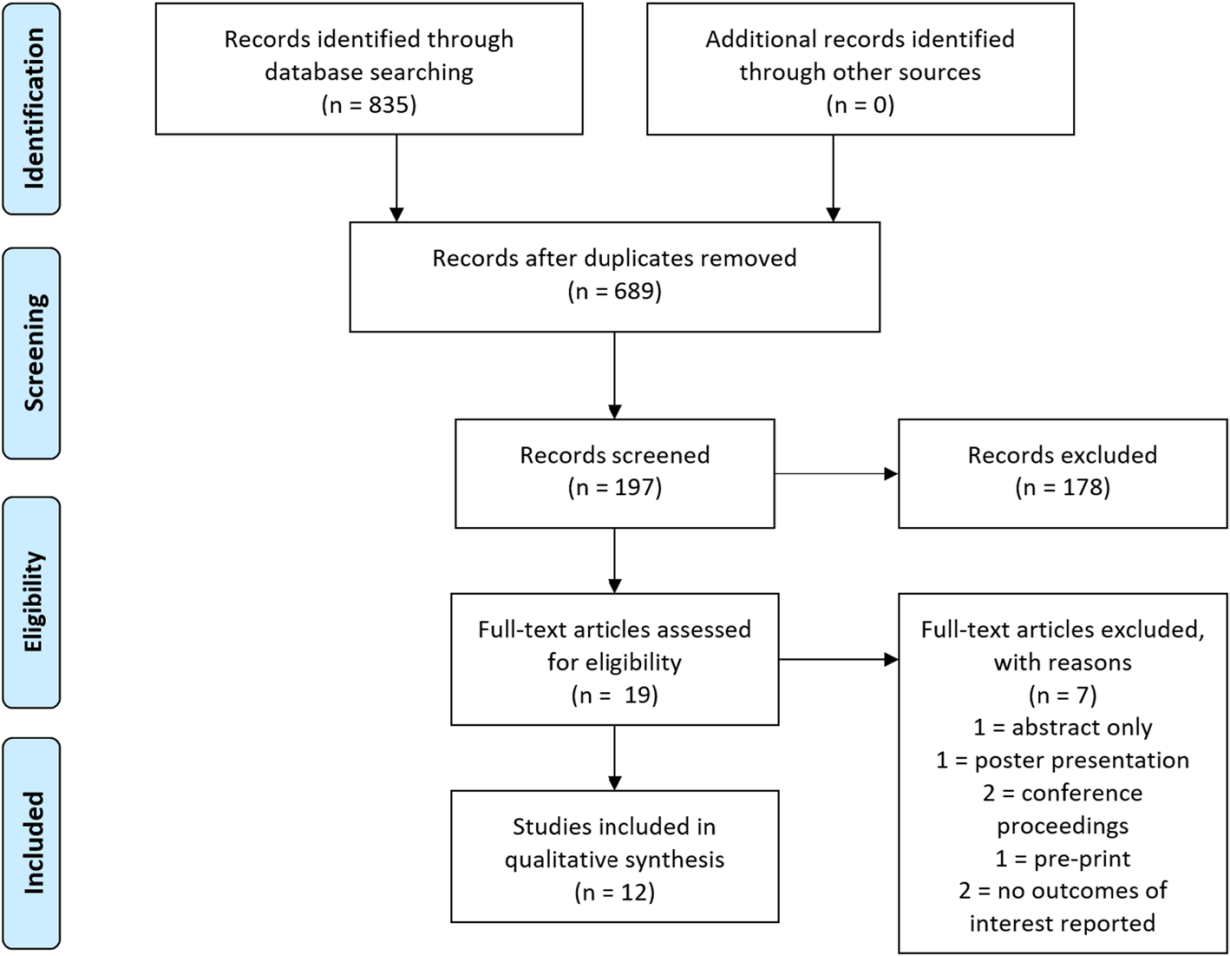
This PRISMA diagram depicts the selection process for article information

PROSPERO protocol registration was performed on July 23rd, 2023 (CRD42023447889). This study followed the Preferred Reporting Items for Systematic Reviews and Meta-Analyses (PRISMA) recommendations [13]. Two assessors, SP and CJH, assessed each study that was returned in our query to determine its eligibility separately. A total of 835 publications were retrieved from the query after duplicates were excluded. After initial screening, 19 were selected for full examination. 12 of these articles satisfied all inclusion criteria. No more articles were found after a thorough review of the reference list of each article. (Fig. 1).

### Study characteristics

Of the 12 studies analyzed, all reported on data from a single institution, with 7 studies being of observational design and 5 cohort studies (Table 1). The sampling frequency of the studies ranged from 44.1 to 64 kHz, with a sampling depth of 16 to 32 bits. A total of 6 studies were about detection of intraoperative fractures, [3, 4, 14–17] while 10 studies investigated the role of acoustic and vibrational analyses on the detection of implant stability and subsidence [2–4, 14–20]. Additionally, 4 studies evaluated the variability of acoustic profiles emitted per implant and patient characteristics [1, 2, 15, 17]. Five studies examined the accuracy of predictive models generated by acoustic analyses [3, 7, 17, 18, 20].

**Table 1:** Characteristics of studies included in the final analysis

Authors (Year)	Study Design	Sample size	Dataset	Sex (% M)	Age	BMI	Implant type	Surgical Approach	Equipment Utilized	Sensor Location	Sampling Frequency	Sampling Depth
Goossens et al. (2020)	Retrospective Cohort	26	Single institution	n/a	20–82 (range)	n/a	Profemur Gladiator	n/a	Microphone: Type 378B02, Software: Simcenter Testlab	n/a	40.96 kHz	n/a
Zhuang et al. (2022) [1, 18]	Retrospective Cohort	55	Single institution	17	66.8 ± 9.8	24.2 ± 3.5	Accolade II	direct anterior	Meter: LA-7500 Software: Oscope v2.1	1 m above ground; 2 m away from surgical table	64 kHz	16 bit
Morohashi et al. (2017) [3]	Prospective Cohort	71	Single institution	16%	65.8 (range 41–86)	24.3 (range 17.7–38.2)	Accolade TMZF	direct lateral: 59 anterolateral:7	Meter: LA-4440 software: LabChart	n/a	40 kHz	n/a
Homma et al. (2022) [7]	Observational	29	Single institution	21%	48–89 (range)	n/a	Accolade II; Taperloc Complete Microplasty; Full-hydroxyapatite porous triple tapered stem	direct anterior	Meter: LA-7500 Software: Oscope v2.1	1 m above ground; 2 m away from surgical table	64 kHz	16 bit
Pastrav et al. (2009) [19]	Observational	83	Single institution	n/a	n/a	n/a	Advanced Custom-Made Implants	n/a	Meter: Pimento vibration analyzer Software: Pimento 5.2	n/a	n/a	n/a
Sakai et al. (2022) [15]	Observational	12	Single institution	33%	70.1	n/a	n/a	n/a	Directional microphone	on the right and left sides of the upper- and lower-limbs of the patient 2 m from the surgical field on each side	44.1 kHz	16 bit
Zhuang et al. (2022) [1, 18]	Retrospective Cohort	49	Single institution	16%	65.7 ± 11.3	24.0 ± 3.69	Taperloc Microplasty; Accolade II	direct anterior	Meter: LA-7500 Software: Oscope v2.1	1 m above ground; 2 m away from surgical table	64 kHz	16 bit
Sakai et al. (2021) [16]	Retrospective Cohort	11	Single institution	36%	71.6 (range 54–81)	n/a	Acetabular cup G7	n/a	Microphone: Mic Fp-5500	n/a	44.1 kHz	16 bit
Homma et al. (2022) [7]	Observational	62	Single institution	17%	66.3 ± 9.6	Height:156.4 ± 7.4 Weight: 58.1 ± 11.1	Accolade II	direct anterior	Meter: LA-7500 Software: Oscope v2.1	1 m above ground; 2 m away from surgical table	64 kHz	16 bit
Homma et al. (2023) [2, 20]	Observational	51	Single institution	19%	68 ± 13	23.19 ± 3.39	Accolade II	direct anterior	Meter: LA-7500 Software: Oscope, v2.1	1 m above ground; 2 m away from surgical table	64 kHz	16 bit
Mulier et al. (2008) [4]	Observational	30	Single institution	40%	53.9 ± 7.6	n/a	Intraoperatively custom made prosthesis stem; Hydroxyapatite coated Pinnacle cup	n/a	Meter: Pimento vibration analyzer software: Pimento 5.2	FRF measured directly on the prosthesis neck	n/a	n/a
McConnell et al. (2018) [17]	Observational	101	Single institution	35%	69.9 ± 12.3	29 (range 26–32)	Corail femoral stem	posterior	Microphone: Auna MRS-01 Software: Amadeus Pro v2.2.2	At the outer edge of the laminar flow canopy	44.1 kHz	32 bit

*BMI* Body mass index, *Hz* Hertz

### Risk of *bias* in individual studies

Two separate reviewers, SP and CJH, utilized the Methodological Index for Nonrandomized Studies (MINORS) tool [21] to appraise bias in the included articles. This validated tool rates articles on a 0 to 24 scale in terms of 12 domains related to study design. For each domain, the ratings are as follows: 0 for not reported, 1 for reported but insufficient, and 2 for documented and sufficient. Y.H., an independent third reviewer, resolved any conflicts over the scoring. The average score was 13.8 ± 0.40.

### Data extraction and analysis

Data regarding research design, acoustic analysis mode, and parameters, as well as intraoperative and postoperative events, were extracted by S.P. and C.J.H.; The results were then compared for verification and any discrepancies were resolved by consulting a third reviewer, Y.H.; As there was a sizable variation in study design across the included publications that would limit the validity of a meta-analysis, we decided to narratively synthesize their main results.

### Ethical approval

All data included in this study were publicly available and did not include protected health information. Therefore, this study was considered exempt from ethical review board approval.

## Results

### Implant stability detection

Two studies involved the use of vibration analysis and the study of frequency response function (FRF) graphs to identify when implant stability is achieved (Table 2). An increased correlation between the FRFs of the last two hammer blows (*r* > 0.99) was noticed in those two studies [4, 19]. One of those studies observed that the higher resonance frequencies were more sensitive to changes in implant stability [19]. This could be emphasized by a right shift in the FRF graph, which denotes increased stability and stiffness. Two studies noted that if the maximum peak frequency stabilized (maximum peak frequency ± 0.5 kHz) on consecutive hammer impactions, hammering should be stopped as the implant stability has been attained and further hammering would cause a fracture [15, 16] Additionally, two studies reported the augmentation and detection of low-frequency bands in the 1 kHz range during the late phase of stem insertion and final femoral broaching [2, 17].

**Table 2:** Key findings from studies evaluating the role of acoustic analysis in detecting implant stability and subsidence

Authors	Methods	Implant Stability and Subsidence
Goossens et al. (2020)	BPF quantifies the relative spectral power distribution of the measured sound signal. PCCs were calculated as a distance metric between the vibroacoustic response spectra of successive insertion hammer blows	18 of 23 implants showed a pattern of increasing BPF and PCCs. BPF best-detected implant seating. A 1 mm subsidence corresponded to approximately a doubling of the BPF value on consecutive hammer blows
Zhuang et al. (2022) [1, 18]	Hammering sounds were compared between hips with and without postoperative subsidence. The frequency spectrum was divided into 25 frequency bands at 0.5 kHz intervals. SP and nSP^a^ were calculated per frequency band	Average subsidence was 2.15 ± 2.91 mm. 9 acoustic parameters were significantly associated with the value of subsidence. The acoustic bands between 5 and 9.5 kHz had significantly lower amounts in hips with subsidence The nSP was higher for those with subsidence in the 0.5 to 2.0 kHz range (divided by 0.5 intervals into 3 sub-groups, all significant)
Morohashi (2017) [3]	Pattern A = frequencies near 7 kHz became more accentuated as implantation progressed (*n* = 42) Pattern B = no accentuation of frequencies near 7 kHz (*n* = 29)	> 1 mm subsidence in 6 of 42 hips in patients with Pattern A and 11 of 27 hips in those with Pattern B, *P* = 0.013
Pastrav et al. (2009) [19]	The FRF changes were used to assess the evolution of the stiffness of the implant-bone structure and, consequently, the progression of the implant stability. When no noticeable change was observed in the FRF graph, hammering was stopped. The similarity of two successive FRF graphs was evaluated using PCCs. A correlation between the FRFs of consecutive stages of R = (0.99 ± 0.01) was considered as the endpoint	A FRF graph right shift indicates increased stiffness and normal evolution between successive hammer blows as reflected by increasing resonance frequencies. Additionally, the higher resonance frequencies are more sensitive to change in implant stability as opposed to the lower resonance frequencies. For non-cemented stems, the FRF between the 4th and 5th hammer blows were correlated > 0.99 for 26/30 cases (86.7%). The other 4/30 were > 0.95 correlation. Therefore, the PCCs between successive FRFs can be used as an endpoint criterion for insertion. For cemented stems, there was a notable difference in FRF between the non-cemented vs. cemented stage (after cement curing) in 85% of these cases (*n* = 45). The shift to the right of the graph indicates an increased stability after cementation. In the remaining 15% (*n* = 8), cement curing did not substantially change the FRF graphs, likely because the stems were already fixed when the cement polymerization was complete
Sakai et al. (2022) [15]	When the maximum peak frequency stays within the range of ± 0.5 kHz three times in a row, the stem was deemed fixed and the miniaturized analysis system provided a warning that further hammering would cause a fracture	In all 12 cases, the system successfully determined stem stability. There was no report of subsidence, implant failure, aseptic loosening, or fracture through a five-year follow-up. The frequency determined at the time of attainment of implant stability was 4.02 ± 2.33 kHz. There were no reports of subsidence in the immediate postoperative period
Sakai et al. (2021) [16]	Stability and cup fixation were defined when the maximum peak frequency changed within ± 0.5 kHz or less in three consecutive blows	The mean stable maximum peak frequency was 4.42 ± 4.02 kHz. A constant maximum peak frequency continued 3.27 ± 0.47 hammering counts. Once the maximum peak frequency stabilizes, as represented by maximum peak frequency ± 0.5 kHz on consecutive blows, hammering should be stopped. There were no cases of immediate postoperative subsidence
Homma et al. (2022) [7]	During final size broaching, the 5 hammering sounds before the last blow were included for analysis	12 out of 62 (19.4%) showed ≥ 3 mm of post-operative subsidence
Homma et al. (2023) [2, 20]	The first three hammering sounds and the final hammering sound were not included to avoid any inconsistencies in hammer impactions. The early phase was defined as the 4th–6th hammering sounds from the beginning. The late phase was defined as the 2nd–4th hammering sounds from the end stem insertion. The frequency spectrum of the hammering sounds was divided into 25 frequency bands at 0.5 kHz intervals. The SP and NSP were assessed for each frequency band. Alternation ratio^b^ was used to define the change in acoustic characteristics	Acoustic characteristics were significantly different between the late phase and the early phase. There was an augmentation of low-frequency bands, as shown by the different alteration ratios of the low-frequency bands (0.5–1 kHz and 1 kHz). For analysis of sound alterations, the low-frequency bands (0.5–1.0 kHz and 1.0–1.5 kHz) were considered the key bands as they showed the most considerable changes
Mulier et al. (2008) [4]	The amount of FRF change between insertion steps was evaluated by calculating PCCs between successive FRFs. A correlation between the FRFs of consecutive stages of R = (0.99 ± 0.01) was considered as the endpoint	In 26 cases (86.7%), the correlation coefficient between the last two FRFs was above 0.99. In the other four cases (13.3%), the surgeon ceased the insertion because of suspected bone fragility
McConnell et al. (2018) [17]	Detection of a low-frequency band centered around 1036 Hz (IQR 944 to 1093) during the final femoral broach represented a ‘change in sound’. This band had not been detected in any of the prior broach impactions	In 75 hips, a "change in sound" was associated with a well-fitted stem (true positive). In 2 hips, a "change in sound" was detected prior to final broaching (false positive). In 24 hips, no change in sound was detected. Out of these 24 hips, 9 hips were judged to have an undersized implant (true negative). The remaining 15 hips in which no change in sound was detected had a well-fitted prosthesis (false negative)

*PCC* Pearson correlation coefficient, *BPF* Band power feature, *Sp* Sound pressure, *NSP* Normalized sound pressure, *FRF* Frequency response function, *IQR* Interquartile range

^a^nSP=SP of each frequency band/total frequency spectrum

^b^Alternation ratio=nSP of late phase/nSP of early phase blows

### Intraoperative fracture detection

According to one study, minimal changes in the FRF graph produced during stem broaching could indicate implant stability (Table 3) [4]. In one case, the study observed an abnormal shape in the FRF graph, in which a minor fracture was immediately found. Another study associated a distinct sound alteration with the occurrence of a femoral fracture: immediately before bone fracture, the frequency and amplitude of the low-frequency band gradually increased and then diminished, the change coinciding with the fracture [17]. One study found that sharp decreases in BPF and PCC during the hammering sequence might serve as a warning for periprosthetic microfracture [14]. Additionally, two articles reported that intraoperative fracture could be prevented by stopping hammering when the peak frequency converges within ± 0.5 kHz during implant fixation across three consecutive blows [15, 16]

**Table 3:** Key findings from studies evaluating the role of acoustic analysis in detecting intraoperative fractures

Authors	Analysis	Intraoperative Fractures Detected	Mode of Fracture Detection	Key Findings
Goossens et al. (2020)	Acoustic	3 (11.5%)	BPF quantifies the relative spectral power distribution of the measured sound signal. PCCs were calculated as a distance metric between the vibroacoustic response spectra of successive insertion hammer blows	A sharp decline in BPF and PCC, by up to approximately 75% during a consistent hammering sequence, suggests implant instability and is a warning for periprosthetic microfracture
Morohashi et al. (2017) [3]	Acoustic	2 (2.8%)	Pattern A = frequencies near 7 kHz became more accentuated as implantation progressed (*n* = 42) Pattern B = no accentuation of frequencies near 7 kHz (*n* = 29)	Intraoperative fracture and postoperative subsidence were less common in patients with Pattern A (*n* = 6) versus Pattern B (*n* = 13) with Pattern B (*P* = 0.004). Both patients with intraoperative fractures displayed Pattern A before fracture and switched to Pattern B immediately after fracture
Sakai et al. (2022) [15]	Acoustic	0 (0%)	When the maximum peak frequency stays within the range of ± 0.5 kHz three times in a row, the stem was deemed fixed and the miniaturized analysis system provided a warning that further hammering would cause a fracture	No fractures were detected. Also, no implants had evidence of aseptic loosening or instability at the five-year follow-up
Sakai et al. (2021) [16]	Acoustic	0 (0%)	Stability and cup fixation were defined when the maximum peak frequency changed within ± 0.5 kHz or less in three consecutive blows	The mean stable maximum peak frequency was 4.42 ± 4.02 kHz. A constant maximum peak frequency continued 3.27 ± 0.47 times. Peak frequency repeats when appropriate fixation is acquired during surgery, suggesting that intraoperative fracture can be prevented by stopping hammering at the time the peak frequency converges within ± 0.5 kHz
Mulier et al (2008) [4]	Vibration	1 (3.3%)	The amount of FRF change between insertion steps was evaluated by calculating PCCs between successive FRFs. A correlation between the FRFs of consecutive stages of R = (0.99 ± 0.01) was considered as the endpoint	Initially, as the stem was partially inserted, the peak of the FRF graph shifted towards frequencies associated with decreased fixation. Further hammering in one case led to an abnormal shape in the FRF graph, in which a small fracture was observed. The FRF's progression can be used to assess implant stability and detect the insertion endpoint. Any variation from the normal evolution of FRF graphs could serve as a warning for impending fracture
McConnell et al. (2018) [17]	Acoustic	1 (1%)	Impaction sounds of the first and last broaches were analyzed to identify prominent frequency bands. In all hips, the frequencies from the initial broach were still present during the impaction of subsequent broaches but at a lower amplitude. Cases were categorized according to the addition of a low-frequency band during subsequent broaching spectrographs contrasted with initial broaching	A low-frequency band was present from the first broach. Subsequent broaching with larger sizes generated a band of gradually increasing frequency and amplitude until the fracture occurred. The one femoral fracture observed brought a distinct sound alteration: immediately before bone fracture, the standing wave progressively gradually increased in frequency and then diminished

*BPF* Band Power Feature, *PCC* Pearson Correlation Coefficient, *N/A* Not applicable, *FRF* Frequency response function

### Factors affecting acoustic analyses

One study observed that the additional low-frequency band originated from inside the femoral canal itself, and thus, the frequency was related to bone length (Table 4) [17]. This finding was corroborated by another study, which revealed an association between the augmentation of low-frequency bands (0.5–1.5 kHz) and stature-related morphological features such as height, weight, and femoral shaft length (FSL) [2]. Another study discerned that femoral morphological features such as Canal-calcar ratio (CCR), Canal-flare index (CFI), Morphologic cortical index (MCI), and FSL influenced hammering sounds in addition to the type of cementless implant used [1]. One study found no association between the recorded frequency and cortical thickness, BMI, or medullary canal diameter [17]. In addition, one study reported that acoustic analysis was more likely to detect hammering sounds at a position near the patient's head as opposed to the left or right side of the body [15].

**Table 4:** Key findings from studies evaluating variance in acoustic profiles emitted per implant and patient characteristics

Authors	Methods	Key Findings
Sakai et al. (2022) [15]	When the maximum peak frequency stays within the range of ± 0.5 kHz three times in a row, the stem was deemed fixed, and the miniaturized analysis system provided a warning that further hammering would cause a fracture	The system was less likely to fail to detect hammering sounds when sounds were collected at a position near the patient's head compared to the left or right side of the body
Zhuang et al. (2022) [1, 18]	The 2nd to 4th hammering sounds from the end were defined as the hammering sounds of the broaching procedure. The frequency spectrum of these sounds was divided into 19 frequency bands, in increments of 0.5 kHz from 0 to 10.0 kHz. Each frequency band was then measured in 2 ways: SP and nSP^a^	In Accolade 2 implants, CCR was positively related to NSP in several bands [Frequency band (kHz); r: 2.0–2.5; 0.37, 4.5–5.0; 0.37, 9.5–10.0; 0.44], and negatively related to 7.5–8.0 kHz (r = − 0.39). Negative correlations were observed among CFI and MCI in specific frequency bands (4.5–5.0, 5.0–5.5, and 7.5–8.0 kHz). In Taperloc Microplasty implants, strong correlations were found between FSL and the NSP of 7.5–8.0 kHz (r = 0.78) and CCR and the 7.5–8.0 kHz bands. Acoustic characteristics of NSPs between Accolade II and Microplasty were different across the 9 frequency bands
Homma et al. (2023) [2, 20]	The alteration ratio^b^ of the 0.5–1.0 kHz frequency band multiplied by the alteration ratio of the 1.0–1.5 kHz frequency band was defined as a feature representing the sound alteration and named the sound alteration value	The augmentation of the low-frequency band (0.5–1.5 kHz) during stem insertion was correlated with stature-related characteristics, such as height, weight, and FSL. On univariate analysis, differences in height, weight, FSL, and CFR2A were associated with different sound profiles emitted. On multivariate analysis, only height and CFR2A were associated with sound alteration values. Height above or below 1.66 m. was identified via decision tree analysis as the single best predictor for the sound alteration value, with > 1.66 m tall patients having the largest sound alteration value
McConnell et al (2018) [17]	Utilizing an acoustic model, in which the femoral canal was considered as a closed-ended hollow tube, the resonant frequency of the femur was estimated, allowing prediction of the fundamental frequency of the standing wave in bone. Additional bands generated during impaction were correlated against femoral length, as well as cortical thickness, medullary diameter, and BMI. Expected frequencies, calculated based on femoral length, were compared with the measured low-frequency bands emitted	A strong correlation was found between the predicted and measured frequency values. No correlations were found between the recorded frequency and cortical thickness (3 cm or 10 cm below greater trochanter), medullary canal diameter, or BMI. Therefore, in cases where an additional low-frequency band was present, there was a strong correlation between predicted and measured frequencies, signifying that frequency is related to bone length

*NSP* Normalized sound pressure, *SP* Sound pressure, *CCR* Canal-calcar ratio, *CFI* Canal-flare index, *MCI* Morphologic cortical index, *FSL* Femoral shaft length, *CFR2A* Canal fill ratio 2 cm above lesser trochanter, *BMI* Body mass index

^a^nSP = SP of each frequency band / total frequency spectrum

^b^Alternation ratio = nSP of late phase/nSP of early phase blows

### Accuracy of predictive models via acoustics

In one study, a model, developed using machine learning techniques, was able to distinguish the final rasping hammering sound with high accuracy (Table 5). Notably, a higher degree of accuracy was noticed with models that analyzed datasets using only 1 implant type (rather than 2 or more). Furthermore, the models performed better at differentiating between acoustic profiles emitted when they were dealing with larger differences in the stem size [7]. Also, another study discussed the innovation of a support vector machine learning algorithm that could predict postoperative subsidence with high accuracy. Adding additional features, such as the patients' basic background features and femoral morphological parameters to nSP (Normalized sound pressure), raised the accuracy of models to nearly 100% [20]. A diagnostic test with a sensitivity of 83.3%, specificity of 81.8%, positive predictive value of 97.4%, and negative predictive value of 37.5% was created using the augmentation of low-frequency bands as an indicator of correctly sized implants [17]. A prediction model for postoperative stem subsidence, as reported in a study in 2022, demonstrated a positive predictive value of 100% and a negative predictive value of 90.6% for postoperative stem subsidence at 5 mm or more [18].

**Table 5:** Key findings from studies evaluating the accuracy of predictive models generated via acoustic analysis

Authors	Methods	Key findings
Zhuang et al. (2022) [1, 18]	Hammering sounds were compared between hips with and without postoperative subsidence. The frequency spectrum was divided into 25 frequency bands at 0.5 kHz intervals. SP and nSP were calculated per frequency band	The post-op subsidence prediction model developed showed a positive prediction value of 100% and a negative prediction value of 90.6% for post-operative stem subsidence at 5 mm or more
Morohashi et al. (2017) [3]	Pattern A = frequencies near 7 kHz became more accentuated as implantation progressed (*n* = 42) Pattern B = no accentuation of frequencies near 7 kHz (*n* = 29)	The sensitivity of Pattern A in predicting a clinical course without adverse events was 69.2% and the specificity was 68.4%. Positive and negative predictive values were 85.7% and 44.8%, respectively. The sensitivity of Pattern B in predicting subsidence was 64.7% and the sensitivity was 69.2%. Positive and negative predictive values were 40.7% and 85.7%, respectively
Homma et al. (2022) [7]	ROC-AUC was used to classify accuracy. Authors adopted input of three types of acoustic profiles into their machine learning algorithm: 1) hammering sound during final size rasping 2) hammering sound during minimum size stem rasping 3) hammering sound during any undersized rasping	Artificial intelligence using machine learning was able to differentiate the final rasping hammering sound from the previous hammering sound. The models had a higher degree of accuracy in analyzing datasets that used only 1 implant type rather than > 1 type utilized. The closer the undersized stem was to the final implanted stem, the less accurate the models were for distinguishing hammering sounds
Homma et al. (2023) [2, 20]	Inputs for models for predicting postoperative subsidence: 1) nSP 2) nSP, patient basic background features 3) nSP, patient basic background features, femoral morphological parameters	The AUC was very high in all models (all > 0.96). Adding additional features such as the patients’ basic background features and femoral morphological parameters to nSP augmented the accuracy of models to nearly 100%
McConnell et al (2018) [17]	Detection of a low-frequency band centered around 1036 Hz (IQR 944 to 1093) during the final femoral broach represented a ‘change in sound’. This band had not been detected in any of the prior broach impactions	As a diagnostic test for correctly sized implants, the change in sound had a sensitivity of 83.3% and a specificity of 81.8%. The positive predictive value was 97.4%, and negative predictive value was 37.5%

*ROC* Receiver Operating Characteristic curve, *AUC* Area under ROC curve, *nSP* Normalized sound pressure, *IQR* Interquartile range

## Discussion

Despite recent advancements with THA, there is a need for a more objective evaluation to assist an orthopedic surgeon in identifying the insertion endpoint of the broaching procedure. A multitude of biomechanical and *in-vitro* studies have demonstrated the promising potential of acoustic and vibration analyses for THA. We conducted this systematic review to evaluate the *in-vivo* efficacy of various acoustic and vibration models. Across methodologies used to evaluate acoustic profiles, analyses have suggested stopping broaching when consecutive blows emit similar acoustic profiles, which indicates proper implant seating for stability and minimizing subsidence. Also, large deviations from the typical progression of acoustic signals while broaching imply that an intraoperative fracture occurred. As patient characteristics and implant-specific parameters have been shown to alter acoustic emissions while hammering, the incorporation of these factors into models to predict subsidence or intraoperative fracture results in increased accuracy in identifying these adverse events.

### Implant stability detection

Augmentation of low-frequency bands (around 1 kHz), stabilization of maximum peak frequency, and increased correlation of FRF during the last two hammer impactions indicate appropriate implant fixation. The quantitative evaluation of the hammering sounds may be a future standard as it is objective, non-invasive, and accurate, as opposed to the auditory sensations of the surgeon, which are subjective [2]. Widespread application of this approach could lead to healthcare savings in addition to successful implantation, as accurate implant sizing mitigates subsidence, migration, and aseptic loosening, which are responsible for practically 50%–60% of revision THAs [22]. One study observed that aseptic loosening within two years was likely due to inadequate implant fixation and poor press fit, with 17% of implant revisions performed before two years [4, 23]. The usage of acoustic analysis may minimize these adverse outcomes after THA, due to its superior detection of implant fixation. Despite the promising results, the use of acoustics for detecting implant stability still needs further study and standardization with respect to methodology, patient population, and acoustic parameters. In addition, the correlation between achieved THA implant stability and clinical outcomes, such as pain relief and functional improvement, should be investigated.

### Intraoperative fracture detection

Current literature indicates that any large deviation from the normal progression of acoustic parameters while hammering might indicate an intraoperative fracture or crack. For instance, sudden attenuation of low-frequency bands in frequency and amplitude implies the occurrence of a fracture [17]. Intraoperative fractures complicate the surgery with prolonged operative time and recovery, inferior outcomes, and an increased risk of revision surgery [24]. Acoustic monitoring may mitigate this burden as surgeons can stop implant impaction when acoustic profiles change. Fractures that go undetected or untreated intraoperatively cause delayed weight bearing, which sometimes requires complicated management strategies and may necessitate complex reoperations. According to a retrospective case series of 6350 THAs, the incidence of undetected intraoperative periprosthetic femoral fractures (IPFFs) was 0.38%, with a reoperation rate of 30.4% [25]. Early recognition of such fractures is integral for optimizing management. While no studies included in this analysis investigated distinctions among different fracture patterns or for acetabular fractures, it has been observed that intraoperative acetabular fractures were rare compared to stem fractures, with an incidence of only 0.4% in cementless cups [26, 27]. Neither did the studies have enough power to evaluate whether surgical approach influenced fracture rates and subsequent acoustic detection, but they have suggested that using minimally invasive or direct anterior approaches during the surgeon’s learning curve are risk factors for intraoperative fracture [28–31]. The application of acoustic analysis can be tailored specifically towards distinguishing fracture patterns to improve rates of detection and better understand the complication profile associated with various techniques.

### Factors affecting acoustic analyses

Sound alteration during broaching is influenced by multiple factors, such as patient stature, femoral morphological characteristics, and implant type. Specifically, one study concluded that particular attention must be paid to people with short stature as they may exhibit a relatively small acoustic change during stem insertion [2]. This may be attributed to the fact that the femur is heavier than the stem, thereby becoming the main vibrating object and the primary source of acoustic emission [2, 17]. In addition, with numerous implant options available to surgeons, the natural frequency and acoustic emittance shapes emitted during broaching are specific for each implant [1–3]. Manufacturers of hip prosthetic implants may elect to report the natural frequency of their implants to assist surgeons in adopting acoustic monitoring during THA. Lastly, further research into how the force of impaction and the resultant slight deformations can lead to different acoustic emissions merits further investigation [32].

### Accuracy of predictive models via acoustics

Although numerous implant and patient-specific parameters influence the variability of acoustic profiles, these limitations can be mitigated by implementing artificial intelligence (AI), machine learning, and other predictive models in sound analysis. Acoustic models using machine learning and AI have demonstrated nearly 100% accuracy in detecting postoperative subsidence [18, 20]. Currently, surgeons subjectively determine when proper implant insertion has been attained through rough auditory and tactile cues. While advances have been made in assisting surgeons in determining the optimal positioning of implants with robotic-assisted and computer-navigated platforms, [33–35] acoustic analyses may offer a similarly more accurate, objective, and non-invasive method to detect proper implant fixation.

### Limitations

This study is not without limitations. The included studies were mostly of an observational nature and did not compare to patient cohorts who underwent THA without acoustic analyses. Likewise, we were unable to control for surgeon experience with THA. Less experienced surgeons may have higher rates of intraoperative fracture and reduced implant stability. Third, there was considerable heterogeneity among studies regarding microphones, sample frequency, spectral analyzer software, and approaches to quantifying the acoustic emissions, leading to difficulty comparing the utility of acoustic analyses across studies. Fourth, many studies had limited sample sizes, as a result, they may have been underpowered to detect the benefits of acoustic emission analyses. Fifth, no studies commented on the relationship between the frequency of blows and implant stress relaxation regarding its possible effect on stability and fracture.

## Conclusions

At present, there is no well-accepted diagnostic tool for predicting implant stability. This review discussed the clinical application of acoustic and vibration analysis during THA. Although studies have demonstrated the benefits of acoustic analyses in detecting proper implant seating and intraoperative fractures and predicting subsidence, further research into the clinical outcomes and long-term implant success in the long run is warranted.

## Data Availability

Data is available upon request to the corresponding author.

## References

[1] Zhuang X, Homma Y, Sato T, et al. Factors influence on the broaching hammering sound during cementless total hip arthroplasty. J Biomed Sci Eng. 2022;15:229–40.

[2] Homma Y, Zhuang X, Yanagisawa N, et al. Patients with shorter stature exhibit minimal hammering sound changes during cementless stem insertion in total hip arthroplasty. Arthroplast Today 21. 10.1016/j.artd.2023.101136. Epub ahead of print 1 June 2023. 10.1016/j.artd.2023.101136 PMC10182171 37193539

[3] Morohashi I, Iwase H, Kanda A, et al. Acoustic pattern evaluation during cementless hip arthroplasty surgery may be a new method for predicting complications. SICOT J 3. 10.1051/sicotj/2016049. Epub ahead of print 2017 10.1051/sicotj/2016049 PMC5302878 28186872

[4] Mulier M, Pastrav C, Van der Perre G. Per-operative vibration analysis: a valuable tool for defining correct stem insertion: preliminary report. Ortop Traumatol Rehabil. 2008;10:576–82. 19153546

[5] Wei JCJ, Crezee WHA, Jongeneel H, et al. Using acoustic vibrations as a method for implant insertion assessment in total hip arthroplasty. Sensors 22. 10.3390/s22041609. Epub ahead of print 1 February 2022 10.3390/s22041609 PMC8877904 35214521

[6] Berend KR, Lombardi AV. Intraoperative Femur Fracture is Associated with Stem and Instrument Design in Primary Total Hip Arthroplasty. Clin Orthop Relat Res. 2010;468:2377–81. 10.1007/s11999-010-1314-8 PMC2919873 20387021

[7] Homma Y, Ito S, Zhuang X, et al. Artificial intelligence for distinguishment of hammering sound in total hip arthroplasty. Sci Rep 12. 10.1038/s41598-022-14006-2. Epub ahead of print 1 December 2022. 10.1038/s41598-022-14006-2 PMC9198079 35701656

[8] Pechon PHM, Pullin R, Eaton MJ, et al. Acoustic emission technology can warn of impending iatrogenic femur fracture during femoral canal preparation for uncemented hip replacement. A cadaveric animal bone study. J Med Eng Technol. 2018;42:72–87. 29560773 10.1080/03091902.2017.1411986

[9] Leuridan S, Goossens Q, Pastrav LC, et al. Development of an instrument to assess the stability of cementless femoral implants using vibration analysis during total hip arthroplasty. IEEE J Transl Eng Health Med 9. 10.1109/JTEHM.2021.3128276. Epub ahead of print 2021. 10.1109/JTEHM.2021.3128276 PMC8791654 35103118

[10] Meneghini RM, Guthrie M, Moore HD, et al. A novel method for prevention of intraoperative fracture in cementless hip arthroplasty: vibration analysis during femoral component insertion. Surg Technol Int. 2010;20:334–9. 21082583

[11] Pérez MA, Seral-García B. A finite element analysis of the vibration behaviour of a cementless hip system. Comput Methods Biomech Biomed Engin. 2013;16:1022–31. 22300407 10.1080/10255842.2011.650635

[12] Whitwell G, Brockett CL, Young S, et al. Spectral analysis of the sound produced during femoral broaching and implant insertion in uncemented total hip arthroplasty. Proc Inst Mech Eng H. 2013;227:175–80. 23513988 10.1177/0954411912462813

[13] Liberati A, Altman DG, Tetzlaff J, et al. The PRISMA statement for reporting systematic reviews and meta-analyses of studies that evaluate healthcare interventions: explanation and elaboration. The BMJ 339. 10.1136/BMJ.B2700. Epub ahead of print 2009. 10.1136/bmj.b2700 PMC2714672 19622552

[14] Goossens Q, Pastrav L, Roosen J, et al. Acoustic analysis to monitor implant seating and early detect fractures in cementless THA: An in vivo study. J Orthop Res. 2021;39:1164–73. 32844506 10.1002/jor.24837

[15] Sakai R. ‘Development of total hip arthroplasty support system for predicting intraoperative fractures by the frequency of hammering sound’. Biomed J Sci Tech Res 44. 10.26717/bjstr.2022.44.007081. Epub ahead of print 20 June 2022.

[16] Sakai R, Uchiyama K, Fukushima K, et al. Hammering sound frequency analysis to fix an acetabular cup during total hip arthroplasty: clinical trials and biomechanical studies. J Biomed Sci Eng. 2021;14:14–20.

[17] McConnell JS, Saunders PRJ, Young SK. The clinical relevance of sound changes produced during cementless hip arthroplasty. Bone Joint J. 2018;100-B:1559–64. 30499313 10.1302/0301-620X.100B12.BJJ-2018-0368.R2

[18] Zhuang X, Homma Y, Ishii S, et al. Acoustic characteristics of broaching procedure for post-operative stem subsidence in cementless total hip arthroplasty. Int Orthop. 2022;46:741–8. 34977970 10.1007/s00264-021-05278-w

[19] Pastrav LC, Jaecques SV, Jonkers I, et al. In vivo evaluation of a vibration analysis technique for the per-operative monitoring of the fixation of hip prostheses. J Orthop Surg Res 4. 10.1186/1749-799X-4-10. Epub ahead of print 2009. 10.1186/1749-799X-4-10 PMC2678089 19358703

[20] Homma Y, Zhuang X, Ohtsu H, et al. Highly accurate acoustical prediction using support vector machine algorithm for post-operative subsidence after cementless total hip arthroplasty. Int Orthop. 2023;47:187–92. 36416898 10.1007/s00264-022-05641-5

[21] Slim K, Nini E, Forestier D, et al. Methodological index for non-randomized studies (MINORS): development and validation of a new instrument. ANZ J Surg. 2003;73:712–6. 12956787 10.1046/j.1445-2197.2003.02748.x

[22] Ries C, Boese CK, Dietrich F, et al. Femoral stem subsidence in cementless total hip arthroplasty: a retrospective single-centre study. Int Orthop. 2019;43:307–14. 29916001 10.1007/s00264-018-4020-x

[23] Feng X, Gu J, Zhou Y. Primary total hip arthroplasty failure: aseptic loosening remains the most common cause of revision. Am J Transl Res. 2022;14:7080–9. 36398241 PMC9641425

[24] Fakler JKM, Brand A, Lycke C, et al. Risk factors for intraoperative greater trochanteric fractures in hemiarthroplasty for intracapsular femoral neck fractures. Eur J Trauma Emerg Surg. 2022;48:1835–40. 33313961 10.1007/s00068-020-01549-0 PMC9192455

[25] Liu Y, Li C, Cao Z, et al. Undetected intraoperative periprosthetic femoral fractures in patients undergoing primary total hip arthroplasty: a retrospective case series and literature review. Orthop Surg. 2023;15:758–65. 36647808 10.1111/os.13646 PMC9977600

[26] Marqués Lopez F, Muñoz Vives JM. Intraoperative periprosthetic hip fractures. Eur Orthop Traumatol. 2013;4:89–92.

[27] Haidukewych GJ. Intraoperative fractures of the acetabulum during primary total hip arthroplasty. J Bone Joint Surg (American). 2006;88:1952. 10.2106/JBJS.E.00890 16951110

[28] Masonis J, Thompson C, Odum S. Safe and accurate: learning the direct anterior total hip arthroplasty. Orthopedics. 2008;31(12 Suppl 2). 19298019

[29] Hartford JM, Knowles SB. Risk factors for perioperative femoral fractures: cementless femoral implants and the direct anterior approach using a fracture table. J Arthroplasty. 2016;31:2013–8. 27084504 10.1016/j.arth.2016.02.045

[30] Asayama I, Kinsey TL, Mahoney OM. Two-year experience using a limited-incision direct lateral approach in total hip arthroplasty. J Arthroplasty. 2006;21:1083–91. 17162165 10.1016/j.arth.2005.09.014

[31] Siddiqi A, Springer BD, Chen AF, et al. Diagnosis and management of intraoperative fractures in primary total hip arthroplasty. J Am Acad Orthop Surg. 10.5435/JAAOS-D-20-00818. Epub ahead of print 20 January 2021. 10.5435/JAAOS-D-20-00818 33475301

[32] Remya AR, Vishwash B, Lee C, et al. Hip implant performance prediction by acoustic emission techniques: a review. Med Biol Eng Comput. 2020;58:1637–50. 32533510 10.1007/s11517-020-02202-z

[33] Chen X, Xiong J, Wang P, et al. Robotic-assisted compared with conventional total hip arthroplasty: systematic review and meta-analysis. Postgrad Med J. 2018;94:335–41. 29776983 10.1136/postgradmedj-2017-135352 PMC5992373

[34] Emara AK, Samuel LT, Acuña AJ, et al. Robotic-arm assisted versus manual total hip arthroplasty: systematic review and meta-analysis of radiographic accuracy. Int J Med Robot Comp Assisted Surg 17. 10.1002/rcs.2332. Epub ahead of print 1 December 2021. 10.1002/rcs.2332 34528372

[35] Kamath AF, Durbhakula SM, Pickering T, et al. Improved accuracy and fewer outliers with a novel CT-free robotic THA system in matched-pair analysis with manual THA. J Robot Surg. 2022;16:905–13. 34709535 10.1007/s11701-021-01315-3 PMC9314281

